# Supplementary material for: Content-rich biological network constructed by mining PubMed abstracts
Source: BMC Bioinformatics. 2004 Oct 8;5:147. doi: 10.1186/1471-2105-5-147 (PMC528731; doi:10.1186/1471-2105-5-147)
Supplement: Additional File 5 — The original Chilibot query results of the term "long-term potentiation (LTP)" and 22 other terms, limiting the latest references analyzed to the years 1990, 1995, 2000, and 2004. [file 1471-2105-5-147-S5.bz2 › chilibotAdditionalFile5/ltp2004/html/CREB_ZIF268.html]

 


 **CREB** and **ZIF268** 
  
Found 6 abstracts in PubMed,  **6 abstracts were retrieved and analyzed**.  


---

 Search Google  |
 PDF files only 
|  EDU domain only 

---

**Interactive relationship** (e.g. stimulation, inhibition, etc)

**Parallel relationship** (e.g. studied together, co-existance, homology, etc.)

- MAPK,  **CREB**  and  **zif268**  are all required for the consolidation of recognition memory.  Ref: 12740127 Philos Trans R Soc Lond B Biol Sci, 2003
- Following a similar time course, the two downstream transcriptional targets of MAPK ERK, cAMP response element binding protein  **CREB**  and the ternary complex factor Elk 1, a key transcriptional regulator of serum response element SRE driven gene expression, were hyperphosphorylated and the immediate early gene  **zif268**  was upregulated.  Ref: 10844026 J Neurosci, 2000
- We show that pharmacological blockade of MAPK ERK phosphorylation, functional inactivation of  **CREB**  in an inducible transgenic mouse and inactivation of  **zif268**  in a mutant mouse result in a similar deficit in long term recognition memory.  Ref: 12740127 Philos Trans R Soc Lond B Biol Sci, 2003
- Here, we briefly review experiments that have shown MAPK ERK, cAMP response element binding protein  **CREB**  and the immediate early gene IEG  **zif268**  are essential components of a signalling cascade required for the expression of late phase LTP and of certain forms of long term memory.  Ref: 12740127 Philos Trans R Soc Lond B Biol Sci, 2003
- Sequencing of the 5 flanking region also revealed potential cis acting elements for multiple transcriptional regulatory factors including Sp1,  **zif268** , Ets,  **CREB** , and PuF sites.  Ref: 9261178 J Biol Chem, 1997
- More recent genetic studies in mice also demonstrated  **CREB**  and inducible transcription factor  **Zif268**  involvement in information storage processes.  Ref: 14515157 Acta Biochim Pol, 2003
